# Association between parity and markers of inflammation: The multi-ethnic study of atherosclerosis

**DOI:** 10.3389/fcvm.2022.922367

**Published:** 2022-09-14

**Authors:** Angelica Ezeigwe, Oluseye Ogunmoroti, Anum S. Minhas, Carla P. Rodriguez, Brigitte Kazzi, Oluwaseun E. Fashanu, Olatokunbo Osibogun, Lara C. Kovell, Colleen M. Harrington, Erin D. Michos

**Affiliations:** ^1^Ciccarone Center for the Prevention of Cardiovascular Disease, Johns Hopkins University School of Medicine, Baltimore, MD, United States; ^2^Division of Cardiology, Sands Constellation Heart Institute, Rochester Regional Health, Rochester, NY, United States; ^3^Department of Epidemiology, Robert Stempel College of Public Health & Social Work, Florida International University, Miami, FL, United States; ^4^Division of Cardiology, University of Massachusetts Chan School of Medicine, Worchester, MA, United States; ^5^Corrigan's Women's Heart Health Program, Massachusetts General Hospital, Boston, MA, United States; ^6^Department of Epidemiology, Johns Hopkins Bloomberg School of Public Health, Baltimore, MD, United States

**Keywords:** parity, inflammation, hsCRP, GlycA, fibrinogen, D-dimer, IL-6, pregnancy

## Abstract

**Introduction:**

Multiparity has been associated with increased risk of cardiovascular disease (CVD). Inflammation may be a mechanism linking parity to CVD. We investigated the association between parity and later-life markers of inflammation.

**Methods:**

We studied 3,454 female MESA participants aged 45–84, free of CVD, who had data on parity and inflammatory markers. Parity was categorized as 0 (reference), 1–2, 3–4, or ≥5. Linear regression was used to evaluate the association between parity and natural log-transformed levels of fibrinogen, D-dimer, GlycA, high sensitivity C-reactive protein (hsCRP), and interleukin-6 (IL-6).

**Results:**

Mean age was 62 ± 10 years. The proportion of women with nulliparity, 1–2, 3–4, and ≥5 live births were 18, 39, 29, and 14%, respectively. There was no association between parity and fibrinogen. Women with grand multiparity (≥5 live births) had 28, 10, and 18% higher levels of hsCRP, IL-6 and D-dimer, respectively, compared to nulliparous women, after adjustment for demographic factors. After additional adjustment for CVD risk factors, women with 1–2 and 3–4 live births had higher hsCRP and women with 1–2 live births had higher GlycA.

**Conclusion:**

In this diverse cohort of middle-to-older aged women, we found that higher parity was associated with some inflammatory markers; however, these associations were largely attenuated after adjustment for CVD risk factors. There was no clear dose-response relationship between parity and these inflammatory markers. Future studies are needed to evaluate how inflammation may influence the link between parity and CVD and whether healthy lifestyle/pharmacotherapies targeting inflammation can reduce CVD risk among multiparous women.

**Clinical trial registration:**

The MESA cohort design is registered at clinicaltrials.gov as follows: https://clinicaltrials.gov/ct2/show/NCT00005487.

## Introduction

Cardiovascular disease (CVD) remains the leading cause of morbidity and mortality in the United States (U.S.) and worldwide (1, 2). CVD is responsible for one-third of the deaths in women worldwide, (1) and in the U.S. about 60 million women have prevalent CVD (1). Unfortunately, heart disease death rates are on the rise in younger and middle-aged women, (3, 4) emphasizing the importance of continued attention to strategies for preventing CVD in women (5, 6). Although there has been some progress including better understanding of some of the underlying pathophysiology of CVD in women, sex and gender disparities in cardiovascular health persist (7). These disparities exist in part due to the underrepresentation of women in previous research studies and the subsequent negative impact on prevention, diagnosis, and treatment for women at risk for CVD (7).

Beyond the traditional risk factors of CVD common in both men and women, evidence suggests that additional sex-based risk factors are important considerations for women (7, 8). These emerging non-traditional risk factors include pregnancy-related conditions such as gestational diabetes and hypertension, preeclampsia and eclampsia (9). Additionally, higher parity (number of live births) has also been shown to be associated with increased risk for future maternal CVD (10–13). A meta-analysis of 10 cohort studies found parity to be independently related to CVD risk with an association between higher number of pregnancies with greater risk of incident maternal CVD (13). Another study found that a history of grand multiparity (≥5 live births) was associated with higher coronary heart disease risk, specifically myocardial infarction, even after adjusting for traditional risk factors (12). A history of grand multiparity has also been found to be associated with worse cardiovascular health (as assessed by the American Heart Association's Life Simple seven metrics) among middle-aged to older women (14). Additionally, when compared to nulliparous women, women with grand multiparity have a higher body mass index (BMI) later in life, (14) an adverse adipokine profile, (15) a more androgenic sex hormone profile, (16) and a greater burden of subclinical atherosclerosis, as assessed by the coronary artery calcium score (17).

The mechanisms linking parity to poorer cardiovascular health are not completely understood. However, the link between inflammation, as measured through several biomarkers, and risk of CVD has been well-documented (18–22). Thus, chronic inflammation may be one mechanism that explain the association between multiparity and increased CVD risk. However, the association of parity with inflammation has been inadequately explored to date.

Our study aims to evaluate the relationship between parity and several markers of inflammation and thrombosis, including high-sensitivity C-reactive protein (hsCRP), interleukin-6 (IL-6), GlycA, fibrinogen, and D-dimer, among middle-aged to older adult women using a multi-ethnic cohort. We hypothesized that increased parity will be associated with higher levels of inflammatory markers.

## Materials and methods

### Study population

The Multi-Ethnic Study of Atherosclerosis (MESA) consists of 6,814 women and men between the ages of 45 and 84 recruited from six study sites across the U.S., free from clinical CVD at the time of enrollment into the study. The study population at the baseline exam consisted of 38% White, 28% Black, 22% Hispanic, and 12% Chinese-American adults, of which 53% were women, all of whom were followed longitudinally to monitor for progression of subclinical CVD. Detailed descriptions of the study population and the conduct of the MESA study have been published elsewhere (23).

For our cross-sectional analysis at the baseline exam, we excluded all men (*n* = 3,213), women with missing parity status (*n* = 2), and those with missing baseline GlycA (*n* = 18), hsCRP (*n* = 18), IL-6 (*n* = 68), D-dimer (*n* = 10), and fibrinogen (*n* = 4) values. We also excluded participants missing observations for other key covariates (*n* = 27) except for pack-years of smoking and current use of menopausal hormone therapy to preserve sample size. Our final analytic sample included 3,454 participants (Supplementary Figure S1).

We obtained approval from Institutional Review Boards at each research center and informed consent from study participants prior to conducting the study.

### Independent variables

Parity (number of live births) and gravidity (total number of pregnancies) were collected by self-report at baseline exam in 2000–2002 and defined based on prior research from the MESA cohort (14, 24). Parity was our primary independent variable for this analysis. Parity was modeled in categories: 0 (nulliparity, reference), 1–2, 3–4, or ≥5 live births, as has been done in previous analyses (14, 25, 26). In a supplemental analysis, we also examined gravidity and inflammation, using similar categories as parity.

### Dependent variables

The dependent variables investigated in this study were the baseline measurements of GlycA, hsCRP, IL-6, D-dimer, and fibrinogen. At the baseline exam, serum levels of inflammatory markers, hsCRP, IL-6, fibrinogen, and D-dimer were measured, as previously reported (27–29). GlycA was measured using nuclear magnetic resonance spectra from EDTA plasma samples stored from the baseline visit. Detailed description on the ascertainment of GlycA measurements in MESA has also been previously described (21, 28, 29).

### Covariates

We included demographic, behavioral, physiologic and CVD risk factors that were measured at the baseline exam from interview questionnaires, medication inventory, physical exam, and fasting laboratory work.

Age (years), BMI (kg/m^2^), systolic blood pressure (mmHg), total cholesterol (mg/dl), and HDL-cholesterol (HDL-C) (mg/dl) were modeled continuously. Race/ethnicity (four groups), study site (six centers), education level (< high school; high school or vocational school; college, graduate, or professional school), smoking status (current/former/never), and menopause status (yes/no) were modeled as categorical variables. Pack-years of smoking and physical activity level (MET-min/week of moderate or vigorous activity) were modeled as continuous variables. Diabetes status (yes/no) was defined as fasting blood glucose ≥126 mg/dl, or non-fasting glucose ≥200 mg/dl or medication use (insulin or oral hypoglycemic medications). The use of lipid-lowering therapy, antihypertensive medications, and menopausal hormone therapy were considered binary variables (yes/no).

### Statistical analyses

We examined baseline characteristics by parity categories. Continuous variables were presented as mean (SD). Categorical variables were presented as frequency (percentages). ANOVA and chi-square statistical tests were used to compare the differences between continuous and categorical variables, respectively.

The inflammatory markers were natural log-transformed in our statistical models to address the skewness of the data. We used progressively adjusted linear regression models to determine the cross-sectional association between parity categories and each of the five inflammatory markers separately. Model 1 adjusted for demographics (age, race/ethnicity) and study site. Model 2 (our primary analytical model) included covariates from model 1 and adjusted for lifestyle and physiologic factors including education, smoking status, pack-years of smoking, physical activity, BMI, menopause status, and current use of menopausal hormone therapy. For model 3, we included all covariates in model 2, plus CVD risk factors and medications, including total cholesterol, HDL-C, use of lipid-lowering medications, systolic blood pressure, use of antihypertensive medications and diabetes status.

The percent difference in the inflammatory markers for the parous groups compared to the reference parity category (i.e., no live births) was calculated from the regression models using the formula, [Exp (β)−1] × 100.

In supplemental analyses, we examined for interactions of parity with obesity (BMI <30 vs. ≥30 kg/m^2^) for the inflammatory markers using the likelihood ratio χ^2^ test in model 2. Additionally, we repeated all models evaluating the association of gravidity (instead of parity) with the inflammatory markers.

Statistical significance was defined at a *p*-value < 0.05. Analyses were performed using STATA Version 16.

## Results

### Baseline characteristics

Out of the 3,454 women included in our study sample, 18% were nulliparous women, 39% had 1–2 live births, 29% had 3–4 live births and 14% had 5 or more live births (Table 1). The overall mean age (SD) of our study population was 62 (10) years, and included 38% White, 28% Black, 22% Hispanic, and 12% Chinese-American women. Mean BMI was 29 (2) kg/m^2^. Women with ≥5 live births were more likely to have higher systolic blood pressure, higher BMI, lower HDL-C, and more likely to have diabetes, as well as slightly higher prevalence of aspirin use (Table 1).

**Table 1:** Baseline characteristics of study participants by parity categories.

	**Total**	**0**	**1–2**	**3–4**	**≥5**	***p*-Value**
	***N*** **=** **3,454**	***n*** **=** **620**	***n*** **=** **1,357**	***n*** **=** **1,004**	***n*** **=** **473**	
Age, years	62 (10)	60 (11)	60 (10)	63 (10)	68 (9)	<0.001
**Race/ethnicity**
White	1,320 (38%)	311 (50%)	541 (40%)	362 (36%)	106 (22%)	<0.001
Chinese-American	412 (12%)	39 (6%)	177 (13%)	145 (14%)	51 (11%)	
Black	971 (28%)	191 (31%)	413 (30%)	248 (25%)	119 (25%)	
Hispanic	751 (22%)	79 (13%)	226 (17%)	249 (25%)	197 (42%)	
**Education**
≥ bachelor's degree	1,029 (30%)	315 (51%)	451 (33%)	235 (23%)	28 (6%)	<0.001
< bachelor's degree	2,425 (70%)	305 (49%)	906 (67%)	769 (77%)	445 (94%)	
**Smoking status**
Never	2,040 (59%)	328 (53%)	754 (56%)	641 (64%)	317 (67%)	<0.001
Former	1,013 (29%)	214 (35%)	425 (31%)	267 (27%)	107 (23%)	
Current	401 (12%)	78 (13%)	178 (13%)	96 (10%)	49 (10%)	
*Pack-years of smoking, if >0	14 (5, 29)	15 (7, 29)	14 (5, 28)	14 (6, 32)	11 (5, 31)	0.66
Physical activity, MET-min/weeks	3,720 (1,832, 6,810)	3,949 (2,010, 6,319)	3,720 (1,875, 6,878)	3,893 (1,983, 7,241)	2,745 (1,118, 6,090)	<0.001
BMI, kg/m^2^	29 (6)	28 (6)	28 (6)	29 (6)	30 (6)	<0.001
**Menopause**
Yes	2,961 (86%)	491 (79%)	1,124 (83%)	895 (89%)	451 (95%)	<0.001
No	493 (14%)	129 (21%)	233 (17%)	109 (11%)	22 (5%)	
**Hormone therapy** ^**†**^
Yes	986 (32%)	179 (33%)	415 (35%)	298 (32%)	94 (21%)	<0.001
No	2,129 (68%)	357 (67%)	786 (65%)	631 (68%)	355 (79%)	
Systolic blood pressure, mmHg	127 (23)	124 (23)	125 (23)	128 (23)	134 (24)	<0.001
Total cholesterol, mg/dl	200 (36)	200 (34)	200 (36)	199 (35)	199 (37)	0.99
HDL-C, mg/dl	56 (15)	59 (16)	57 (15)	55 (15)	53 (13)	<0.001
Diabetes	389 (11%)	46 (7%)	146 (11%)	118 (12%)	79 (17%)	<0.001
Antihypertensive medication	1,308 (38%)	196 (32%)	498 (37%)	400 (40%)	214 (45%)	<0.001
Lipid-lowering medication	565 (16%)	82 (13%)	231 (17%)	172 (17%)	80 (17%)	0.14
NSAIDS excluding Aspirin	740 (21%)	143 (23%)	308 (23%)	194 (19%)	95 (20%)	0.14
Aspirin^‡^	550 (17%)	95 (16%)	187 (14%)	178 (18%)	90 (20%)	0.02
GlycA, umol/L	390 (351, 435)	383 (344, 424)	393 (351, 439)	391 (352, 436)	390 (355, 434)	<0.01
CRP, mg/L	2.5 (1.0, 5.6)	2.1 (0.9, 4.5)	2.6 (1.0, 6.0)	2.6 (1.1, 5.6)	3.0 (1.3, 5.9)	<0.001
IL-6, pg/ml	1.3 (0.8, 1.9)	1.2 (0.8, 1.8)	1.2 (0.8, 1.9)	1.3 (0.9, 1.9)	1.6 (1.0, 2.3)	<0.001
Fibrinogen, mg/dl	352 (308, 403)	347 (305, 402)	346 (300, 398)	358 (313, 406)	368 (327, 412)	<0.001
D-dimer, μg/ml	0.2 (0.2, 0.4)	0.2 (0.1, 0.4)	0.2 (0.1, 0.4)	0.3 (0.2, 0.4)	0.3 (0.2, 0.6)	<0.001

BMI, body mass index; CRP, c-reactive protein; HDL-C, high-density lipoprotein-cholesterol; IL-6, interleukin-6; MET, metabolic equivalent of task; NSAIDS, non-steroidal anti-inflammatory drugs.

Data were presented as mean (SD), median (IQR) or number (percentage).

*N = 3,422 for pack-years of smoking; ^†^N = 3,115 for hormone therapy; ^‡^N = 3,300 for Aspirin.

### Inflammatory markers

The association between parity categories and log-transformed inflammatory markers are displayed in Table 2.

**Table 2:** Association between parity and inflammatory markers in MESA.

**Parity**	***N***	**Model 1, *N* = 3,454**	**Model 2, *N* = 3,087**	**Model 3, *N* = 3,087**
		**Percent difference (95% CI)**		
**GlycA**
0	620	Reference	Reference	Reference
1–2	1,357	**3 (1, 4)**	**2 (1, 4)**	**2 (1, 4)**
3–4	1,004	**2 (1, 4)**	1 (0, 3)	1 (0, 3)
≥5	473	1 (−1, 3)	−1 (−3, 1)	−1 (−3, 1)
**CRP**
0	620	Reference	Reference	Reference
1–2	1,357	**25 (12, 39)**	**18 (6, 30)**	**18 (7, 31)**
3–4	1,004	**27 (13, 43)**	**17 (4, 30)**	**16 (4, 30)**
≥5	473	**28 (11, 48)**	12 (−2, 29)	12 (−3, 28)
**IL-6**
0	620	Reference	Reference	Reference
1–2	1,357	−4 (−9, 2)	−3 (−8, 3)	−2 (−8, 3)
3–4	1,004	2 (−5, 8)	1 (−5, 7)	0 (−6, 7)
≥5	473	**10 (1, 19)**	1 (−7, 9)	0 (−7, 8)
**Fibrinogen**
0	620	Reference	Reference	Reference
1–2	1,357	−1 (−2, 1)	−1 (−3, 1)	−1 (−3, 1)
3–4	1,004	1 (−1, 3)	0 (−2, 3)	0 (−2, 2)
≥5	473	0 (−2, 3)	−2 (−4, 1)	−2 (−4, 1)
**D-dimer**
0	620	Reference	Reference	Reference
1–2	1,357	1 (−6, 10)	1 (−7, 10)	1 (−7, 10)
3–4	1,004	7 (−2, 17)	5 (−4, 15)	5 (−4, 15)
≥5	473	**18 (6, 31)**	10 (−2, 23)	10 (−2, 23)

CRP, c-reactive protein; IL-6, interleukin-6; MESA, multi-ethnic study of atherosclerosis.

Results were presented as percent difference calculated from [exp (β)−1] × 100 for the association between parity and natural log-transformed inflammatory markers.

Reference = 0 (nulliparous).

Statistical significant results at p < 0.05 are in bold font.

Model 1 (demographics and study site): age, race/ethnicity and study site.

Model 2 (model 1 + lifestyle and physiologic factors): education, smoking status, pack-years of smoking, physical activity, BMI, menopause status and current use of menopausal hormone therapy.

Model 3 (model 2 + CVD risk factors and medications): total cholesterol, HDL-C, lipid-lowering medication, systolic blood pressure, antihypertensive medication and diabetes.

For GlycA, a history of 1–2 live births was associated with higher levels of GlycA across all three adjusted models, compared to nulliparity. However, for women with 3–4 live births, a significant difference for higher GlycA was observed only after adjustment for demographic factors (model 1) and was attenuated after further adjustment. There was no statically significant difference for grand-multiparity (i.e., ≥5 live births) with GlycA when compared to the reference in any of the three models.

For hsCRP, women with a history of 1–2 live births and 3–4 live births had higher levels compared to nulliparous women in all three adjusted models. After full adjustment for all CVD risk factors (model 3), women with 1–2 live births and 3–4 live births had 18 and 16% higher hsCRP levels, respectively, compared to nulliparous women. Women with ≥5 live births had higher hsCRP levels in the demographic adjusted model (model 1) only.

**Figure 1:**
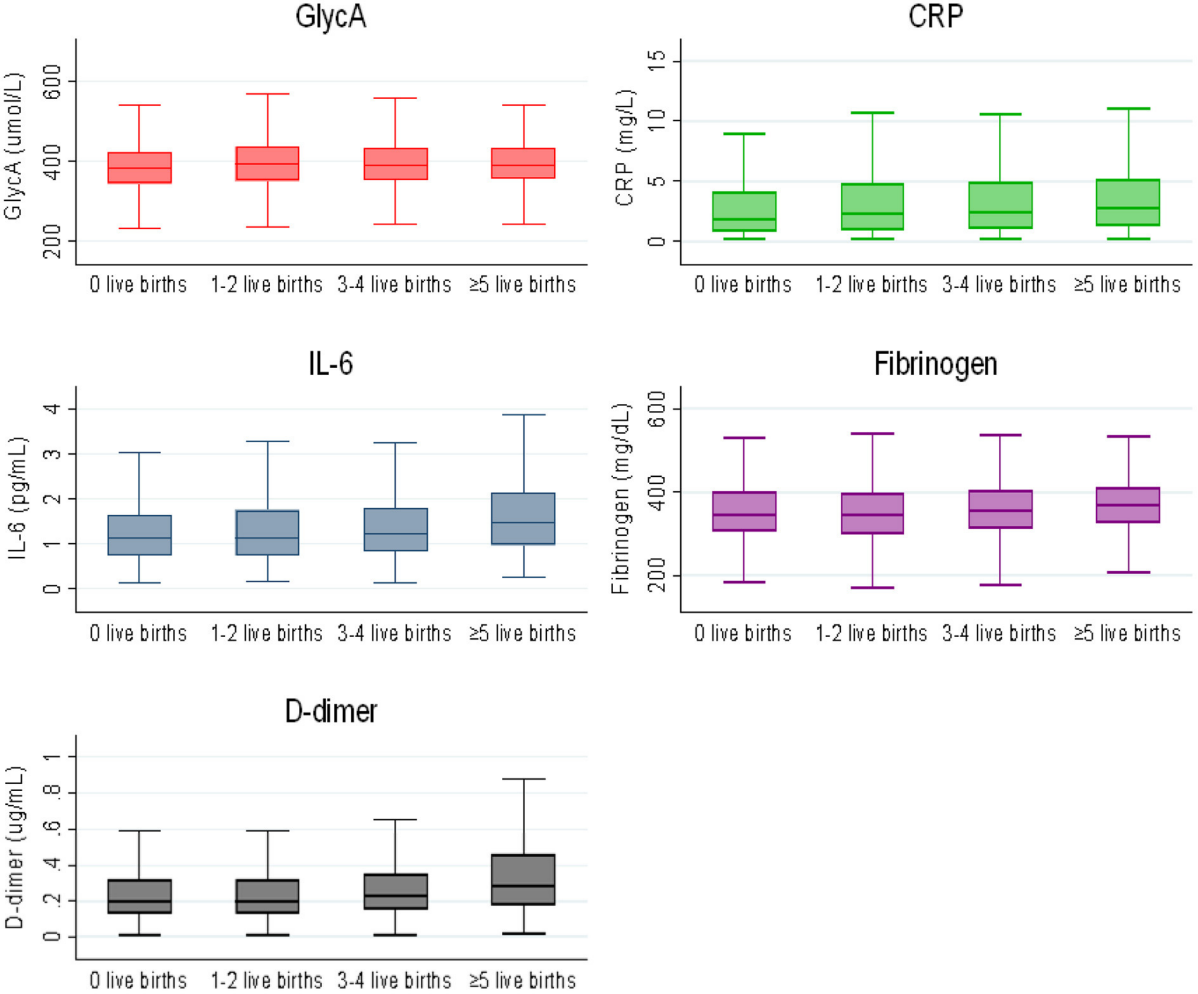
Box plot of inflammatory markers by parity categories. The lower and upper boundaries of the rectangles denote the 25th and 75th percentiles while the horizontal line within the rectangles is the median. Lines extend from the rectangles to the smallest and largest values within 1.5 × interquartile range.

For IL-6 and D-dimer, women with a history ≥5 live births were found to have higher levels in the unadjusted (Figure 1) and demographic adjusted models (model 1) only. There was no significant association of parity with fibrinogen.

In a supplemental analysis, we found no statistically significant interaction of parity with BMI on any of the inflammatory markers (*p* > 0.05).

Additional supplemental analysis for gravidity (Supplementary Table S1) showed that out of the 3,454 women included in our study sample, 13% were nulligravida women, 32% had 1–2 pregnancies, 33% had 3–4 pregnancies and 23% had 5 or more pregnancies. Multigravida women were more likely to be Hispanic, have higher systolic blood pressure, higher BMI, lower HDL-C and more likely to have diabetes (Supplementary Table S1).

The association between gravidity categories and log-transformed inflammatory markers are displayed in Supplementary Table S2. For GlycA, a history of 1–2 pregnancies was associated with higher levels of GlycA for model 1 alone, compared to nulligravida.

For hsCRP, women with a history of 1–2 pregnancies and 3–4 pregnancies had higher levels compared to nulligravida women in all three adjusted models. After adjustment for all CVD risk factors (model 3), women with 1–2 pregnancies and 3–4 live births had 21 and 18% higher hsCRP levels, respectively, compared to women who had no pregnancy history. Women with a history of ≥5 pregnancies were found to have 25% higher hsCRP and 11% higher D-dimer levels in the demographic adjusted model (model 1) only compared to nulligravida women, which was no longer statistically significant after further covariate adjustment. There were no significant associations between gravidity and IL-6 or fibrinogen across all models and gravidity categories, compared to the nulligravida group.

## Discussion

In this multi-ethnic cohort of women who were free of CVD at initial time of assessment, we found that a history of higher parity was positively associated with some inflammatory markers, though these finding did not always remain statistically significant with in fully adjusted models. Specifically, we found that after accounting for differences in age, race/ethnicity, lifestyle and physiologic factors, as well as CVD risk factors and medication use, women with a history of 1–2 live births were found to have higher levels of GlycA and hsCRP compared to nulliparous women. Women with a history of 3–4 live births also had higher hsCRP levels compared to women with no live births. Women with grand multiparity (≥5 live births) had higher levels of hsCRP, IL-6, and D-dimer in demographic adjusted models, but this was attenuated and no longer statistically significant after adjustment for CVD risk factors. Thus, there was no clear dose-response relationship between parity and inflammatory levels.

Nevertheless, we did find that the associations of parity and inflammation were strongest for hsCRP. Our findings are comparable with another prior cross-sectional study of Mexican-American women, which also found parity to be associated with elevated CRP (30). Another cross-sectional analysis showed that the inflammatory marker, IL-12, was elevated in those increasing parity (categorized as 0, 1, 2, 3, and 4 or more pregnancies), although the relationship was not significant after adjusting for smoking (31). These studies, plus our findings, suggest that parity may be more closely related to certain inflammatory markers.

Women tend to gain weight on average with each subsequent pregnancy, and prior work in MESA confirmed that women with grand multiparity had higher BMIs compared to other parous groups (14). Adipokines (hormones secreted by adipose tissue) play a role in both normal and abnormal pregnancies, (32) and adipokine dysregulation may be one mechanism by which pregnancy-associated weight changes may confer later life maternal CVD risk (15). It is well-established that adiposity leads to a pro-inflammatory state (33). Although we hypothesized that greater parity would be independently associated with inflammatory markers, perhaps it is not surprising that indeed for several inflammatory markers (i.e., IL-6 and D-dimer), associations between grand multiparity were attenuated and no longer significant after accounting for BMI in model 2. However, parity did remain associated with hsCRP even in fully adjusted models, including BMI, at least for 1–2 and 3–4 live births. Our analysis did not reveal an influence of BMI on the association between parity and inflammation. In prior studies, higher BMI has been associated with chronic inflammation and adipose tissue has been shown to release pro-inflammatory cytokines (34, 35). Thus, the association between parity and inflammation may be mediated by another factor not evaluated in our study.

We found similar findings for gravidity, with the strongest association observed with hsCRP. The discrepancies in association using gravidity in comparison to parity, lies in the confounder that women with higher gravidity may or may not have been successful in completing their pregnancy for several reason, which may confer different risk for CVD.

### Strengths and limitations

Our study was meant to be exploratory to determine if there was a link between parity history and later life inflammatory risk in women. However, our study findings should be considered in the context of several limitations. First, it is an observational study; therefore, causality cannot be inferred; residual confounding may explain some of the associations seen. Additionally, with a cross-sectional analysis, it is prone to temporal and survival bias. Women in our study had a mean age of 62 and were predominantly menopausal; thus, the inflammatory markers were measured on average a significant number of years from the women's last pregnancies, and unfortunately, we did not have the age at last pregnancy available to determine the time lag. Using the average menopause age for U.S. women, of 51 years, there are at least 11 years on average from last pregnancy at the time of the study. Third, we may also have adjusted for some mediators between parity and inflammation such as BMI that led to attenuation of the relationship between parity and inflammation. Fourth, there was a smaller sample size for the grand multiparous group, which may have contributed to less statistical power to detect a significant difference. Additionally, a key confounder in multiparity is social class, which was adjusted for by considering two categories of education and may result in residual confounding. Finally, there was no information collected in MESA about adverse pregnancy outcomes such as pre-eclampsia, gestational diabetes or preterm birth, so we could not examine a history of these high-risk pregnancy conditions with later life inflammatory markers.

The strengths of our study include the use of a multi-ethnic cohort of women who were free of CVD at baseline. We adjusted for numerous confounders in our models for the relationship between parity and inflammation. This study contributes to the currently under-explored area in understanding the potential mechanism in which higher parity may be contributing to poorer cardiovascular outcomes in women. To our knowledge, our study was the first to explore the association of parity with later life elevations of other markers of inflammation and thrombosis (i.e., GlycA, IL-6, fibrinogen, and D-dimer).

## Conclusion

In this diverse cohort of mid-life to older-aged women free from clinical CVD, we found a history of higher parity was positively associated with some inflammatory markers; however, these associations were largely attenuated after adjustment for lifestyle and CVD risk factors. There was no clear dose-response relationship between higher parity status and higher inflammatory levels. Future studies are needed to evaluate how other markers of inflammation may influence the link between parity and CVD and whether lifestyle/pharmacotherapy targeting inflammation can reduce CVD risk among multiparous women.

## Data availability statement

The datasets presented in this article are not readily available but datasets can be made available by request to the MESA Publications Committee after signing Data Transfer Agreement or by submitting a request to NIH NHBLI BioLincc at https://biolincc.nhlbi.nih.gov/studies/mesa//. Requests to access the datasets should be directed to https://biolincc.nhlbi.nih.gov/studies/mesa/.

## Ethics statement

The studies involving human participants were reviewed and approved by Johns Hopkins University Institutional Review Board. The patients/participants provided their written informed consent to participate in this study.

## Author contributions

AE and EM designed the study. AE performed statistical analysis under supervision of OOg. AE wrote the first draft under EM mentorship. AM, OOs, OF, CR, BK, LK, and CH provided critical scientific input to manuscript draft. All authors approved of the final submission.

## Funding

The MESA study was supported by contracts HHSN268201500003I, N01-HC-95159, N01-HC-95160, N01-HC-95161, N01-HC-95162, N01-HC-95163, N01-HC-95164, N01-HC-95165, N01-HC-95166, N01-HC-95167, N01-HC-95168, and N01-HC-95169 from the National Heart, Lung, and Blood Institute (NHLBI), and by grants UL1-TR-000040, UL1-TR-001079, and UL1-TR-001420 from the National Center for Advancing Translational Sciences. EM is additionally funded by the Amato Fund for Women's Cardiovascular Health at Johns Hopkins University.

## Conflict of interest

Unrelated to this work, Author EM has served on advisory boards for Pfizer, Esperion, Novartis, Novo Nordisk, Bayer, Boehringer Ingelheim, Amarin, and Astra Zeneca.

The remaining authors declare that the research was conducted in the absence of any commercial or financial relationships that could be construed as a potential conflict of interest.

## Publisher's note

All claims expressed in this article are solely those of the authors and do not necessarily represent those of their affiliated organizations, or those of the publisher, the editors and the reviewers. Any product that may be evaluated in this article, or claim that may be made by its manufacturer, is not guaranteed or endorsed by the publisher.
