# Supplementary material for: Association between parity and markers of inflammation: The multi-ethnic study of atherosclerosis
Source: Front Cardiovasc Med. 2022 Sep 14;9:922367. doi: 10.3389/fcvm.2022.922367 (PMC9515387; doi:10.3389/fcvm.2022.922367)
Supplement: Supplementary file 1 [file Table_1.docx]

| **Table S1. Baseline characteristics of study participants by gravidity categories** | | | | | | |
| --- | --- | --- | --- | --- | --- | --- |
|  | Total | 0 | 1-2 | 3-4 | ≥5 | p value |
|  | N = 3,454 | n = 432 | n = 1,093 | n = 1,146 | n = 783 |  |
| Age, years | 62 (10) | 61 (11) | 61 (10) | 62 (10) | 65 (10) | <0.001 |
| Race/ethnicity |  |  |  |  |  |  |
| White | 1,320 (38%) | 228 (53%) | 469 (43%) | 435 (38%) | 188 (24%) |  |
| Chinese-American | 412 (12%) | 33 (8%) | 96 (9%) | 163 (14%) | 120 (15%) | <0.001 |
| Black | 971 (28%) | 108 (25%) | 356 (33%) | 305 (27%) | 202 (26%) |  |
| Hispanic | 751 (22%) | 63 (15%) | 172 (16%) | 243 (21%) | 273 (35%) |  |
| Education |  |  |  |  |  |  |
| ≥ bachelor's degree | 1,029 (30%) | 227 (53%) | 378 (35%) | 322 (28%) | 102 (13%) | <0.001 |
| <bachelor’s degree | 2,425 (70%) | 205 (47%) | 715 (65%) | 824 (72%) | 681 (87%) |  |
| Smoking status |  |  |  |  |  |  |
| Never | 2,040 (59%) | 252 (58%) | 595 (54%) | 683 (60%) | 510 (65%) |  |
| Former | 1,013 (29%) | 140 (32%) | 348 (32%) | 338 (29%) | 187 (24%) | <0.001 |
| Current | 401 (12%) | 40 (9%) | 150 (14%) | 125 (11%) | 86 (11%) |  |
| Pack-years of smoking, if >0 | 14 (5, 29) | 15 (8, 29) | 15 (5, 30) | 14 (6, 29) | 12 (5, 27) | 0.93 |
| Physical activity, MET-min/wk | 3720  (1833, 6810) | 4043  (2099, 6214) | 3765 (1935, 6885) | 3810  (1980, 7118) | 3390  (1380, 6585) | <0.001 |
| BMI, kg/m^2^ | 29 (6) | 28 (6) | 29 (6) | 29 (6) | 29 (6) | 0.001 |
| Menopause |  |  |  |  |  |  |
| Yes | 2,961 (86%) | 344 (79%) | 922 (84%) | 979 (85%) | 716 (91%) | <0.001 |
| No | 493 (14%) | 88 (20%) | 171 (16%) | 167 (15%) | 67 (9%) |  |
| Hormone therapy |  |  |  |  |  |  |
| Yes | 986 (32%) | 127 (34%) | 343 (35%) | 337 (33%) | 179 (25%) | <0.001 |
| No | 2,129 (68%) | 245 (66%) | 643 (65%) | 692 (67%) | 549 (75%) |  |
| Systolic blood pressure, mmHg | 127 (23) | 124 (23) | 126 (23) | 126 (23) | 131 (24) | <0.001 |
| Total cholesterol, mg/dL | 200 (36) | 201 (33) | 201 (36) | 200 (36) | 197 (35) | 0.33 |
| HDL-C, mg/dL | 56 (15) | 58 (16) | 58 (16) | 56 (15) | 53 (14) | <0.001 |
| Diabetes | 389 (11%) | 28 (6%) | 121 (11%) | 121 (11%) | 119 (15%) | <0.001 |
| Antihypertensive medication | 1,308 (38%) | 131 (30%) | 413 (38%) | 433 (38%) | 331 (42%) | 0.001 |
| Lipid-lowering medication | 565 (16%) | 54 (13%) | 189 (17%) | 191 (17%) | 131 (17%) | 0.14 |
| GlycA, umol/L | 390  (351, 435) | 384  (346, 424) | 397  (354, 440) | 389  (350, 436) | 389  (352, 432) | <0.01 |
| CRP, mg/L | 2.5  (1.0, 5.6) | 1.8  (0.9, 4.3) | 2.7  (1.1, 6.1) | 2.6  (1.0, 5.7) | 2.7  (1.1, 5.6) | <0.001 |
| IL-6, pg/mL | 1.3 (0.8, 1.9) | 1.2 (0.7, 1.8) | 1.2 (0.8, 1.9) | 1.2 (0.8, 1.9) | 1.5 (0.9, 2.2) | <0.001 |
| Fibrinogen, mg/dL | 352  (308, 403) | 347  (305, 397) | 347  (304, 404) | 353  (308, 400) | 362  (319, 407) | <0.01 |
| D-dimer, ug/mL | 0.2 (0.2, 0.4) | 0.2 (0.1, 0.4) | 0.2 (0.1, 0.4) | 0.2 (0.2, 0.4) | 0.3 (0.2, 0.5) | <0.001 |
| Abbreviations: BMI, body mass index; CRP, c-reactive protein; HDL-C, high-density lipoprotein-cholesterol; IL-6, interleukin-6; MET; metabolic equivalent of task.  Data were presented as mean (SD), median (IQR) or number (percentage).  *N=3,422 for pack-years of smoking; †N=3,115 for hormone therapy. | | | | | | |
