# Supplementary material for: Association between parity and markers of inflammation: The multi-ethnic study of atherosclerosis
Source: Front Cardiovasc Med. 2022 Sep 14;9:922367. doi: 10.3389/fcvm.2022.922367 (PMC9515387; doi:10.3389/fcvm.2022.922367)
Supplement: Supplementary file 2 [file Table_2.docx]

| **Table S2. Association between gravidity and inflammatory markers in MESA** | | | | |
| --- | --- | --- | --- | --- |
|  |  | Model 1, N =3,454 | Model 2, N=3,087 | Model 3, N=3,087 |
|  | N | Percent difference (95% CI) | | |
| Gravidity | GlycA | | | |
| 0 | 432 | Reference | Reference | Reference |
| 1-2 | 1,093 | **3 (1, 5)** | **2 (0, 4)** | **2 (0, 4)** |
| 3-4 | 1,146 | 2 (0, 3) | 1 (-1, 2) | 1 (-1, 2) |
| ≥5 | 783 | 1 (-1, 3) | -1 (-2, 1) | 0 (-2, 1) |
|  | CRP | | | |
| 0 | 432 | Reference | Reference | Reference |
| 1-2 | 1,093 | **25 (10, 41)** | **19 (5, 34)** | **21 (7, 36)** |
| 3-4 | 1,146 | **26 (11, 43)** | **17 (3, 32)** | **18 (4, 33)** |
| ≥5 | 783 | **25 (9, 43)** | 12 (-2, 28) | 13 (-1, 29) |
|  | IL-6 | | | |
| 0 | 432 | Reference | Reference | Reference |
| 1-2 | 1,093 | -1 (-8, 6) | -1 (-8, 6) | 0 (-7, 7) |
| 3-4 | 1,146 | 1 (-6, 8) | -1 (-7, 6) | -1 (-7, 6) |
| ≥5 | 783 | 7 (-1, 16) | 2 (-5, 10) | 1 (-6, 9) |
|  | Fibrinogen | | | |
| 0 | 432 | Reference | Reference | Reference |
| 1-2 | 1,093 | 0 (-3, 2) | -1 (-3, 1) | -1 (-3, 1) |
| 3-4 | 1,146 | 0 (-2, 2) | 0 (-2, 2) | 0 (-2, 2) |
| ≥5 | 783 | -1 (-3, 2) | -2 (-4, 1) | -2 (-4, 1) |
|  | D-dimer | | | |
| 0 | 432 | Reference | Reference | Reference |
| 1-2 | 1,093 | -2 (-11, 7) | -2 (-12, 8) | -2 (-11, 8) |
| 3-4 | 1,146 | 5 (-4, 15) | 3 (-6, 14) | 4 (-6, 15) |
| ≥5 | 783 | **11 (0, 23)** | 9 (-2, 21) | 9 (-2, 22) |
| Abbreviations; CRP, c-reactive protein; IL-6, interleunkin-6; MESA, Multi-Ethnic Study of Atherosclerosis.  Results were presented as percent difference calculated from [Exp (β) -1]*100 for the association between parity and natural log-transformed inflammatory markers.  Reference = 0 (nulliparous)  Statistical significant results at p<0.05 are in bold font  Model 1 (demographics and study site): age, race/ethnicity and study site.  Model 2 (model 1 + lifestyle and physiologic factors): education, smoking status, pack-years of smoking, physical activity, BMI, menopause status and current use of menopausal hormone therapy.  Model 3 (model 2 + CVD risk factors and medications): total cholesterol, HDL-C, lipid-lowering medication, systolic blood pressure, antihypertensive medication and diabetes. | | | | |
