# Supplementary material for: Association between parity and markers of inflammation: The multi-ethnic study of atherosclerosis
Source: Front Cardiovasc Med. 2022 Sep 14;9:922367. doi: 10.3389/fcvm.2022.922367 (PMC9515387; doi:10.3389/fcvm.2022.922367)
Supplement: Supplementary file 3 [file Image_1.pdf]

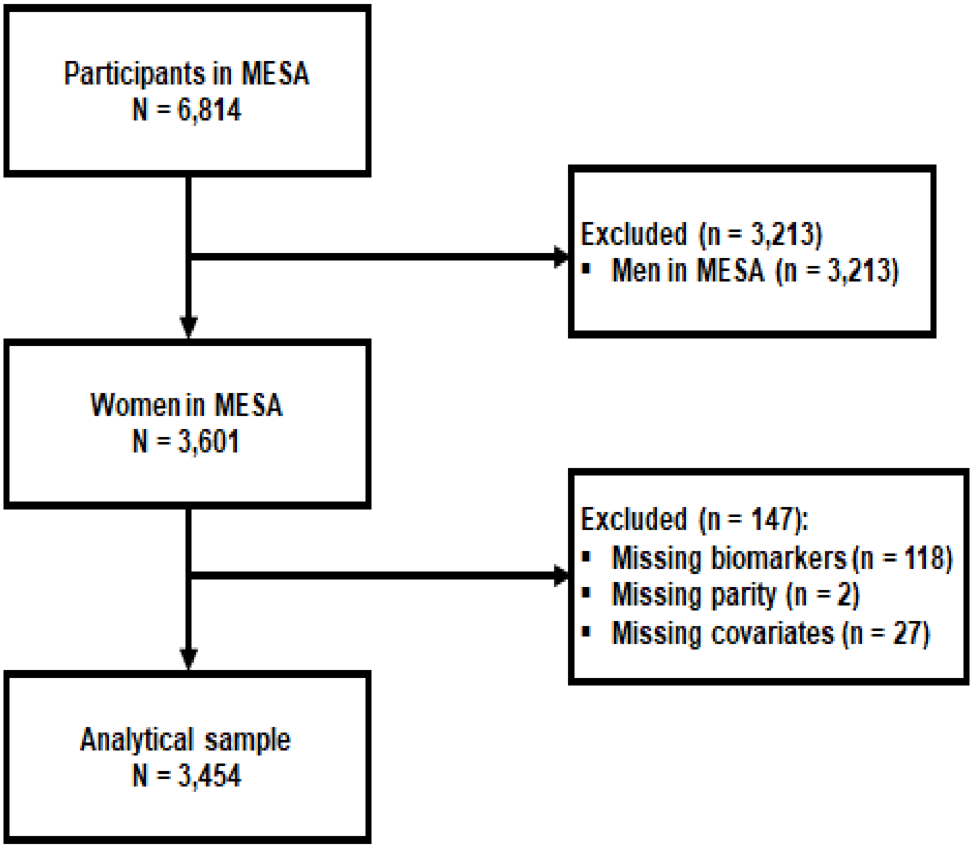

Figure S1. Flowchart of study participants

To preserve sample size, we did not exclude missing observations for pack-years of smoking, n = 32 and hormone therapy, n = 339.
